# Chemical Composition of an Anthelmintic Fraction of *Pleurotus eryngii* against Eggs and Infective Larvae (L3) of *Haemonchus contortus*

**DOI:** 10.1155/2020/4138950

**Published:** 2020-08-06

**Authors:** Julio Cruz-Arévalo, José E. Sánchez, Manases González-Cortázar, Alejandro Zamilpa, Rene H. Andrade-Gallegos, Pedro Mendoza-de-Gives, Liliana Aguilar-Marcelino

**Affiliations:** ^1^El Colegio de la Frontera Sur, Carretera al Antiguo Aeropuerto km. 2.5 C.P, 30700 Tapachula, Chiapas, Mexico; ^2^Centro de Investigación Biomédica del Sur, Instituto Mexicano del Seguro Social (CIBIS-IMSS), Argentina No. 1. Col. Centro, Xochitepec, Morelos C.P. 62790, Mexico; ^3^Centro Nacional de Investigación Disciplinaria en Salud Animal e Inocuidad, INIFAP, Km 11 Carretera Federal Cuernavaca-Cuautla, No. 8534, Col. Progreso, Morelos, Jiutepec, CP 62550, Mexico

## Abstract

This study was aimed at evaluating the *in vitro* effect of the edible mushroom (EM) *Pleurotus eryngii* against the eggs and larvae (L3) of *Haemonchus contortus*. The evaluation included acetone (AE) and hydroalcoholic (HA) extracts of the following strains: ECS-1138, ECS-1156, ECS-1255, ECS-1258, ECS-1261, ECS-1282, and ECS-1292. The HA extract of the ECS-1255 strain showed the highest effect on mortality rates of L3 (18.83%) at 20 *μ*g/mL. After subjecting this HA extract to a normal phase chromatography column, five fractions were obtained; fraction F5 (100% MeOH) was the most effective against eggs, with hatching inhibition percentages of 88.77 and 91.87% at 20 and 40 mg/mL, respectively. Gas chromatography-mass spectrometry (GC-MS) subjected this fraction to an acetylation reaction to determine the content of the secondary metabolites. The GC-MS analysis showed that the F5 fraction was composed of trehalose CAS: 6138-23-4, polyols (L-iditol CAS: 488-45-9, galactitol CAS: 608-66-2, D-mannitol CAS: 69-65-8, D-glucitol CAS: 50-70-4, and myoinositol CAS: 87-89-8), adipic acid CAS: 124-04-9, stearic acid CAS: 57-11-4, squalene CAS: 111-02-4, and *β*-sitosterol CAS: 83-46-5.

## 1. Introduction

The nematode *Haemonchus contortus* is a gastrointestinal parasite that affects small ruminants and causes major economic losses worldwide [1]. Infection begins when sheep ingest the infective larvae (L3) of nematodes present in grass dew, which provides optimum moisture conditions for the growth and survival of these parasites [2]. At each stage of their lifecycle, the high degree of genetic adaptability allows them to transcribe many genes related to biological needs and environmental conditions, while their high prolificacy ensures the survival of large numbers of L3 larvae [3]. In Mexico, the main method adopted by sheep farmers to control *H. contortus* is chemical treatment [4]. However, frequent and excessive nematicidal doses have caused these parasites to develop anthelmintic resistance, which is hereditary. Therefore, different alternatives have been implemented to control the populations of gastrointestinal nematodes in sheep [5]. Several nematotoxic compounds have been isolated and identified in edible mushrooms (EM), such as fatty acids, alkaloids, peptide compounds, terpenes [6], condensed tannins, phenolic compounds [7], and proteases [8]. Until 2013, 23 species of the *Pleurotus* genus mushroom had shown nematicidal activity (NTA), which is considered a characteristic of this genus [9]. *Pleurotus eryngii* is an EM described as a “complex species” due to the significant variations in the morphology, isozymes, and genetic characteristics between specimens. These variations are derived from geographical and ecological differences in their environment [10]. The NTA of *P. eryngii* have been observed against *Syphacia obvelata* and the cestode *Hymenolepis nana*, and it reaches population reductions of 95 and 89%, respectively [11]. A study by Braga et al. [12] reported in vitro mortality rates of 47.56% against *Ancylostoma caninum* larvae. Li et al. [13], Mamiya et al. [14], and Palizi et al. [15] have studied the NTA of *P. eryngii* against free-living and plant parasitic nematodes; however, there are few reports on the effectiveness against *H. contortus*. Thus, the present study was aimed at evaluating the NTA of seven strains of *P. eryngii* and identified the active compounds against eggs and L3 larvae of *H. contortus* by GC-MS.

## 2. Materials and Methods

### 2.1. Harvest of Basidiomata

The seven *P. eryngii* strains used in this study (Table 1) were grown on a sterile substrate (a mixture of corncob, grass, and sawdust, 65% moisture) according to Sánchez and Royse [16]. Basidiomata were produced at El Colegio de la Frontera Sur (ECOSUR), located in Tapachula, Chiapas, Mexico. Fruiting bodies were harvested when the pileus reached maximum extension, were cut longitudinally, and were subjected to natural drying in the shade.

### 2.2. Collection, Chemical Fractionation, and Mycochemical Characterization of Extracts

The collection, fractionation, and mycochemical characterization of the extracts by thin-layer chromatography (TLC) were carried out in the Phytochemical Department of Centro de Investigación Biomédica del Sur (CIBIS) of the Instituto Mexicano del Seguro Social (IMSS) located in Xochitepec, Morelos, Mexico.

#### 2.2.1. Collection of Acetonic Extracts

Dried mushrooms were crushed manually and soaked in acetone (≥99.5%, ACS) at a 10 : 1 ratio (solvent : fruiting bodies). The same mushrooms were soaked at three time points (24, 72, and 96 h) reaching three extracts at the different time points. Each maceration product was filtered through a filter paper membrane and then concentrated on a rotary evaporator (Laborota 4000, 45°C/900 mbar/80 RPM) to obtain the remaining concentration, which was dried with a freeze dryer (Heto Drywinner) to obtain a lyophilized sample that was considered an acetonic extract (AE). Subsequently, an aliquot of AE was dissolved in 10 mL of dichloromethane and was subjected to a qualitative TLC analysis. Aluminium slides precoated with silica gel 60 F_254_ and a mobile phase (95 : 5, *v*/*v*) prepared with dichloromethane (≥99.5%, ACS) : methanol (≥99.8%, ACS) were used [17]. The spots were observed by short- and long-wave UV light and developed with Ce(SO_4_)_2_, and ursolic acid purchased from Sigma-Aldrich (≥90%) was used as the reference standard.

#### 2.2.2. Collection of Hydroalcoholic Extracts

The dried mushrooms were submerged under ethanol- (96%) distilled water solution (60 : 40, *v*/*v*) for 24 h, at the same ratio as that of AE. The liquid obtained was filtered and concentrated in a rotary evaporator (55°C/900 mbar). The remaining fraction was lyophilized to obtain the dry hydroalcoholic extract (HA). An aliquot was dissolved in 10 mL of MeOH and was subjected to TLC analysis; the mobile phase and chemical developer were *α*-naphthol (≥99%) and Ce(SO_4_)_2_, and the reference compounds were oleanolic acid (≥93.22%) and *β*-sitosterol-D-glucoside (≥75.0%). Both were purchased from Sigma-Aldrich, and the spots were also visualized by short- and long-wave UV light.

#### 2.2.3. Column Fractionation of Hydroalcoholic Extract of ECS-1255 Strain

The HA from the ECS-1255 strain showed the highest degree of NTA, and it was fractionated using open-column chromatography. A chromatographic column was packed up with 35 g of normal-phase silica gel (70–230 mesh, Merck). The extract (3.6 g) was adsorbed on 5 g of normal-phase silica and added to the column. Fractionation was performed by three eluent dichloromethane-methanol systems (CH_2_Cl_2_-MeOH: 90 : 10, 70 : 30, 50 : 50 *v*/*v*), and the column was washed with MeOH J.T. Baker® (≥99.8%). Four fractions of 250 mL were collected from each system (a total of 1 L). However, a precipitate was formed during the last collection from the 70 : 30 system, which was also considered as a fraction; thus, five fractions (F1–F5) were collected. Each eluted fraction was concentrated with the same parameters used for AE, then lyophilized, and stored in glass vials. TLC analysis of the F5 fraction dissolved in MeOH was performed using a butanol- (99.4%) acetic acid- (≥98%) distilled water system (3 : 5 : 2 *v*/*v*), and the spots were stained with *α*-naphthol and visualized by UV light.

### 2.3. *In Vitro* Bioassays

The egg and larvae collection and the *in vitro* bioassays were performed at the Instituto Nacional de Investigaciones Forestales, Agrícolas y Pecuarias (INIFAP), in the Department of Helminthology of the Centro de Investigación Disciplinaria en Salud Animal e Inocuidad (CENID-SAI), located in Jiutepec, Morelos, Mexico.

#### 2.3.1. Collection of L3 Larvae and Eggs of *H. contortus*

Prior to infection with infecting larvae, the McMaster technique was performed to verify that the sheep was not infected with any eggs of another nematode. Subsequently, the sheep was dewormed with commercial Ivermectin Iverfull **®** (IVM) 200 *μ*g/kg live weight. A healthy male sheep (3 months) was infected with *H. contortus* larvae at a dose of 350 L3/kg live weight according to Pineda-Alegría et al. [18]. After a prepatent period of 21 days, faeces were collected directly from the rectum of infected animals, and the McMaster technique was performed to observe the presence of nematode eggs and estimate the number of eggs per g of faeces. A stool culture with adequate moisture (28°C) and ventilation was prepared in a basin at room temperature. After seven days, L3 larvae were recovered using the Baermann larval migration technique and washed, and then the sheath was removed according to the methods described by Liébano-Hernández et al. [19]. The eggs were obtained by taking a stool sample directly from the rectum of the sheep. The eggs were subsequently purified according to the technique used by Pineda-Alegría et al. [18]. The treatment of the sheep was carried out following Mexican standards (NOM-051-ZOO-1995 and LEY FEDERAL DE SALUD ANIMAL).

#### 2.3.2. Assessment of *P. eryngii* Extracts against L3 Larvae of *H. contortus*

*In vitro* confrontation was performed five times according to the indications in Table 1. The confrontation mixture between the extract and 100 L3 larvae was placed in 0.2 mL microtubes and stirred with a vortex to ensure homogeneity. The tubes were covered and incubated for 72 h at 28 ± 1°C. The NTA was measured by placing 10 *μ*L of aliquots from the mixture on a slide and counting dead larvae using a compound microscope with 10x objective. The criterion for distinguishing live and dead larvae was motility and response to physical stimuli.

The NTA effectiveness was calculated using the following formula:

(1) $$\begin{array}{c} \%\text{ of mortality}=\left(\left(a‒b\right)/\left(100‒b\right)\right)\times 100, \end{array}$$

where *a* is the mean of the treatment and *b* is the mean of the negative control, according to Belemlilga-Bonewendé et al. [20].

#### 2.3.3. *In Vitro* Evaluation of the F5 Fraction against Eggs and L3 Larvae of *H. contortus*

A previous bioassay (data not shown) of the F1–F5 fractions against L3 larvae was performed to evaluate which was more effective. It was shown that the F5 fraction had better results. *In vitro* bioassays against eggs and larvae were carried out on ELISA plates. The concentrations were adjusted starting from a stock solution of F5 fraction at 80 mg/mL dissolved in MeOH at 8%. The final concentrations of F5 fraction were 3, 5, 10, 20, and 40 mg/mL against eggs and larvae. The mixtures were prepared with 50 *μ*L of the respective treatment and 50 *μ*L of an aqueous suspension containing an average of 100 *H. contortus* larvae or 100 *H. contortus* eggs. For the assay on larvae, a negative control (MeOH) at 4% and a positive control at 5 mg/mL were used. For the test on eggs, a negative control (DMSO) at 2.5% and a positive control, IVM at 5 mg/mL, were used. The mixtures were kept at 28 ± 1°C for 72 h. The egg hatching inhibition test was made by taking aliquots of 10 *μ*L from the mixtures, and the formation of the L1 larvae was observed using a compound microscope (10x) [18]. The hatching inhibition percentage was determined by the same formula used in the extract bioassays.

#### 2.3.4. Statistical Analysis

The mortality and inhibition effectiveness were compared using a completely randomized ANOVA LSD test for extract confrontation and Tukey's test for the mean comparison of the F5 fraction (*α* = 0.05) using “R” statistical software version 3.2.1 (2016). In addition, the median lethal concentration (LC50) of the F5 fraction was determined by probit analysis fitted to a generalized linear model.

### 2.4. Derivatization of the F5 Fraction

The F5 fraction was subjected to an acetylation reaction according to Kitson et al. [21] with some modifications. Three millilitres of acetic anhydride was added to 10 mg of F5. One millilitre of pyridine was added as a catalyser, and after 15 min, the reaction was stopped with ice. The crude reaction mixture was supplemented with ethyl acetate, which yielded two phases. The fraction containing the compounds (FRAcet) was concentrated in a rotary evaporator (45°C/900 mbar/80 RPM). Subsequently, the acetylated fraction was absorbed on silica gel (70–320 mesh) and eluted with CH_2_Cl_2_ 100% and CH_2_Cl_2_ : MeOH (90 : 10) systems on a glass column with normal-phase silica gel. The two subfractions (EXT94 and EXT95) were sent to the “Centro de Investigaciones Químicas de la Universidad Autónoma del Estado de Morelos” (CIQ-UAEM) for analysis by gas chromatography-mass spectrometry (GC-MS).

### 2.5. GC-MS Analysis of F5 Fraction

GC-MS analysis of the EXT94 and EXT95 subfractions was performed using an Agilent 6890 gas chromatograph interfaced to an Agilent 5973N Mass Selective Detector with ionization energy (70 eV). An HP-5MS column (30 m × 0.250 mm id., 0.25 *μ*m film thickness, coated with 5% diphenyl and 95% dimethylpolysiloxane) was used. Helium gas (99.99%) was used as the carrier gas at a constant flow rate of 1 mL/min, and an injection volume of 1 *μ*L was employed. The temperature of the injector was 250°C, and the ion source temperature was 230°C. The oven temperature was kept at 40°C for 1 min, and then the temperature was programmed at 5°C/min to 250°C, which was maintained for 1 min. Finally, the temperature was increased 10°C/min to 285°C and was kept for 20 min.

## 3. Results

### 3.1. Mycochemical Profile of Extracts and F5 Fraction

The compounds present in both extracts were qualitatively analysed by TLC. The chemical reaction with Ce(SO_4_)_2_ suggests the presence of several phenolic compounds in the AE performed with CH_2_Cl_2_-MeOH (95 : 5). In the case of the HA extracts, most of the compounds did not migrate with the mobile-phase CH_2_Cl_2_-MeOH (95 : 5); however, spraying the slides with Ce(SO_4_)_2_ showed that the profiles of the strains aligned with the migration profile of oleanolic acid. The Rf value of oleanolic acid was 0.57, while the value profile of the HA extracts was 0.60. The presence of glucosidic compounds was observed as well as possible terpenes, which shared a profile with *β*-sitosterol glucoside.

### 3.2. Observing the Anthelmintic Effect of Extracts

The NTA of HA was more effective than AE. The ECS-1255 strain was the only strain that showed activity in both extracts (Table 2), and the difference was statistically significant compared to the other strains of the AE group; however, this value was lower compared to IVM. Regarding the HA extracts, the ECS-1255 strain had the greatest NTA with 18.83%, although there was no significant difference compared with strains ECS-1258, ECS-1156, and ECS-1292, and the effect was significantly lower than the control with IVM (*p* ≤ 0.0001).

### 3.3. Effect of the F5 Fraction against Eggs and Larvae

**Figure 1:**
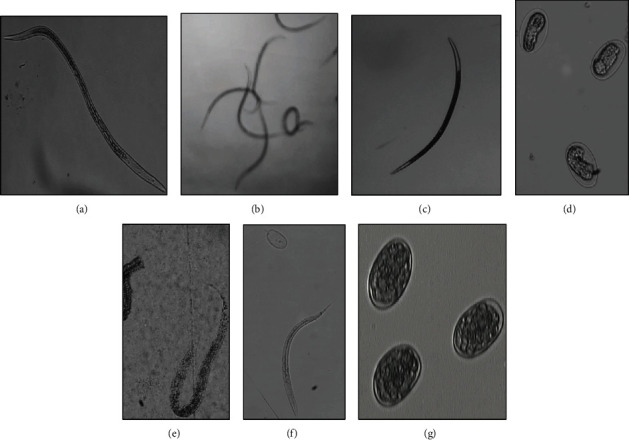
Eggs and larvae damaged by the F5 fraction. (a) Larva with internal ulcers. (b) Active larvae in the negative control. (c) Dead larva in the positive control (IVM). (d) Larval development inhibited in the eggs. (e) A dead L1 larva that hatched but did not survive. (f) A L1 larva that hatched in the negative control. (g) Egg hatching inhibited by positive control (IVM).

The F5 fraction showed no effect on larval mortality (data not shown); however, the larvae confronted with this fraction showed little motility and presented internal ulcers and contractions (Figure 1(a)) compared with the negative control larvae, which were very active and without physical damage (Figure 1(b)). In the egg confrontation test, the egg hatching inhibition percentages ranged from 42.78 to 91.87% for the above mentioned concentrations (Table 3). Several eggs and L1 larvae were completely distorted in the treatments with the highest concentrations (Figures 1(d) and 1(e)), while the negative control L1 larvae hatched (Figure 1(f)). There was no statistically significant difference between the effectiveness of the positive control IVM and the F5 fraction at concentrations of 40 and 20 mg/mL. The calculated LC50 was 4.19 mg/mL, and the upper and lower confidence limits were 4.60 and 3.12 mg/mL, respectively.

### 3.4. Compounds Identified in the F5 Fraction by GC-MS

**Figure 2:**
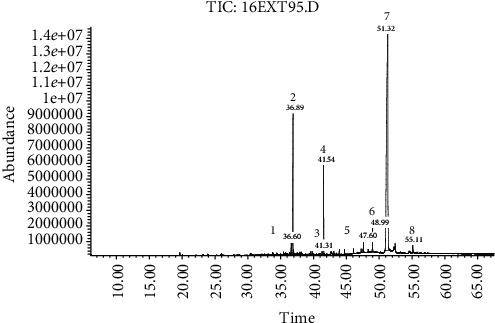
GC-MS of the first subfraction. Peaks: (1) myoinositol, (2) D-glucitol, (3) stearic acid, (4) adipic acid, (5) squalene, (6) nd (not detected), (7) trehalose, and (8) *β*-sitosterol.

**Figure 3:**
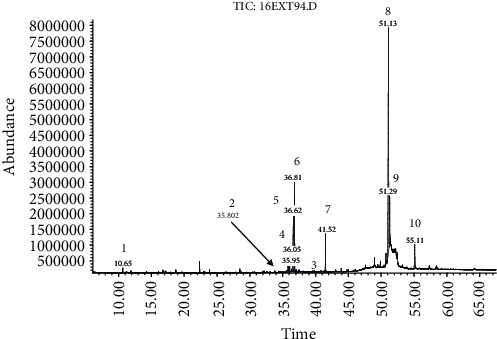
GC-MS of the second subfraction. Peaks: (1) propanol, (2) galactitol, (3) L-iditol, (4) L-iditol, (5) D-glucitol, (6) mannitol, (7) adipic acid, (8) trehalose, (9) trehalose, and (10) *β*-sitosterol.

Interpretation of the mass spectrum was conducted using the database of the National Institute Standard and Technology (NIST Version 1.7). The time retention of identified compounds is shown in Figures 2 and 3. The compounds in the EXT95 subfraction were trehalose CAS: 6138-23-4 (73.36%), polyols (D-glucitol CAS: 50-70-4, and myoinositol CAS: 87-89-8) (17%), adipic acid CAS: 124-04-9 (6.33%), squalene CAS: 111-02-4 (0.41%), stearic acid CAS: 57-11-4 (0.25%), and *β*-sitosterol CAS: 83-46-5 (1.02%). In the EXT94 subfraction, the mixture consisted of 65.71% trehalose, while polyols (L-iditol CAS: 488-45-9, galactitol CAS: 608-66-2, D-mannitol CAS: 69-65-8, and D-glucitol) accounted for 24.20%, *β*-sitosterol for 5.05%, and adipic acid for 4.22%.

## 4. Discussion

The *in vitro* NTA of extracts obtained from the mycelium of *P. eryngii* against *H. contortus* L3 have already been evaluated; however, the effect of basidiomata extracts is unknown. In a study by Comans-Pérez [22], the HA mycelium extracts from the *P. eryngii* ECS-1292 strain caused 95% L3 mortality after 48 h of *in vitro* confrontation. The efficiency percentage of the ECS-1255 strain is similar to the *in vitro* study reported by Huicochea-Medina [23] who evaluated 400 *μ*g/mL of HA extracts from *P. eryngii* mycelium (ECS-1292 strain) against L3. After 24 h of confrontation, a mortality rate of 16.6% was observed. The similarity between these results indicates that the biological state of the mushroom influences the number of nematicidal metabolites present in the extract. It is presumed that higher concentrations of carpophore extract yield better mortality rates. Moreover, it is possible that drying the mushroom influenced the presence of metabolites. A number of flavour components varied when Li et al. [24] evaluated the *P. eryngii* mushrooms under different drying methods. HA extracts caused physical damage to the larvae. They had internal ulcers in their body, which were probably caused by HA extract absorption through transcuticular diffusion. HA extracts from *Croton macrostachyus* seeds and *Ekebergia capensis* and *Hedera helix* plants showed NTA values between 60 and 100% against *H. contortus* eggs and larvae [25, 26]. These authors attribute the effect to the ease of transcuticular absorption of the HA extracts; furthermore, they mention that most of anthelmintic drugs enter the body of nematodes, tapeworms, and trematodes through this pathway. Harder [27] indicates that *H. contortus* absorbs nematicidal drugs, such as levamisole and macrocyclic lactones, through the cuticle. The NTA of HA extracts has also been observed against phytonematodes. Oka [28] obtained the best NTA against *Meloidogyne javanica* with aqueous HA extracts from *Verbesina encelioides* flowers. This suggests that HA extraction is a good alternative for obtaining efficient nematicidal metabolites.

The high percentages of egg-hatching inhibition indicate that the F5 fraction from the *P. eryngii* ECS-1255 strain has nematicidal compounds. The variety of glucide compounds present in the fraction suggests that they could be responsible for the ovicidal effect. According to Oka [28], glucoside compounds have nematicidal properties. However, there is no certainty that trehalose, which is the major component of the fraction, is responsible for the NTA; in fact, this sugar is necessary for the survival of nematodes under water stress and freezing conditions [29]. Trehalose also plays a fundamental role during larval development within the egg and the physiology of the nematodes [30]. Although it has been observed that trehalose can inhibit egg hatching by osmotic pressure, this effect is temporary and reversible, since expelling trehalose through the permeable membrane allows the larvae to continue their development.

Five polyols were identified in the present study. Four of them have aliphatic structure, which is very similar to xylitol, while myoinositol is a cyclic polyol. It is likely that these polyols are responsible for the ovicidal effect. Compounds with analogous structures to nematicidal molecules might have some nematotoxic effect. According to Marie-Magdeleine et al. [31], the nematicidal properties of the cucurbitin amino acid are due to the similarity of its structure with the kainic acid NTA. Seo et al. [32] observed high NTA of natural ester compounds and their analogues against *B. xylophilus*. The embryonic development of the silkworm *Bombyx mori* was inhibited by sorbitol (D-glucitol) polyol; interestingly, trehalose also played a detrimental role in embryonic development [33].

An older study showed that the egg hatching of *M. incognita* was inhibited by 23% with a synthetic polyol over the semisolid phase; moreover, some juvenile (J2) larvae were found distorted [34]. In another survey, Cedillo-Rodríguez [35] obtained 100% hatching inhibition in *H. contortus* eggs exposed *in vitro* to a fraction containing 1.25 mg/mL of xylitol. The bioactive properties of this polyol are known to have antimicrobial effects and benefits for dental health [36]. Moreover, Nilsson et al. [37] mentioned that xylitol polyol could inhibit children's ear infection. In general, polyols are compounds with several hydroxyl functional groups; Park et al. [38] reported the stronger NTA of compounds with hydroxyl or methoxy groups against *B. xylophilus* nematode. This could support the hypothesis that polyols are principal bioactive compounds against *H. contortus* eggs in the present study.

The *β*-sitosterol was also detected in the F5 fraction. This compound can be conjugated with sugars, so this sterol may have formed complexes with glucide carbohydrates in the F5 fraction and thus acts synergistically. For example, the glucoside *β*-sitosterol had a maximum effective concentration (EC50) of 82.50 *μ*g/mL when used to challenge the nematode *Caenorhabditis elegans* [39]. In a revision by Ohri and Pannu [40], it was noted that glycoside compounds vary in their mortality rates on the *M. incognita* nematode. Oka [28] mentioned that glycosides are known for their nematicidal properties.

Hrckova and Velebny [41] also documented the NTA of glycoside compounds. According to Park et al. [38], several alcohols, fatty acid derivatives, aldehydes, terpenoids, and phenolic compounds can act synergistically or independently as insecticidal or nematicidal agents. Conjugation of sugars may also explain the poor migration of compounds on silica plates and the chromatographic separation.

Stearic acid was also detected in the F5 fraction. The NTA of fatty acids is already known. According to Degenkolb and Vilcinskas [42], linoleic acid is noted as acting in a nematicidal fashion against nematophagous trap-forming fungi. The NTA of stearic acid has been observed against *C. elegans* [43], *M. incognita*, *Aphelenchoides besseyi*, and *Panagrellus redivivus* [44]. The structure of adipic acid is also very similar to that of fatty acids; however, according to Nagase et al. [45], it had no effect when it was evaluated against *B. lignicolous*. Therefore, the NTA against *H. contortus* is unlikely.

Squalene is a triterpene that was also identified in the F5 fraction. Terpenes are widely known for their NTA [40]. The bioactive properties of squalene have been studied in terms of their cytotoxic and antimalarial effects [46]. Seven different triterpenes caused mortality rates between 10 and 80% in *M. incognita* [47]. Terpenes, such as ursolic acid and *β*-sitosterol, showed 70 and 60% effectiveness when confronted against *M. incognita* phytonematodes [48]. However, although squalene is a triterpene, its nematicidal effect was not observed in the present study when the squalene standard was confronted against *H. contortus* L3 larvae (data not shown).

Since there were no significant differences between the concentrations of 20 and 40 mg/mL and IVM, the F5 fraction might be considered as an alternative nematicidal that should be evaluated *in vivo* models. According to the standards of the WAAP (World Association for the Advancement of Veterinary Parasitology) for rating anthelmintics, a 90% efficacy is considered very good, while an 80–90% efficacy is moderately effective [49].

## 5. Conclusions

Unlike other species of the *Pleurotus* genus, the nematotoxic compounds of *P. eryngii* had not yet been identified. This study demonstrates the presence of several compounds in *P. eryngii*, whose nematicidal effect had been previously reported for other genera. Furthermore, we identified compounds with no previous reports of NTA but with similar structures to natural or synthetic nematicidal compounds that could be considered for evaluation as an alternative nematicidal. In the present study, five different polyols were identified, whereas only three have been typically reported in strains of the *Pleurotus* genus. The assessment of polyols identified (individually or in combinations) as inhibitors of *H. contortus* egg hatching can possibly be considered for the control of this parasite. Successful results could make the *P. eryngii* mushroom a potential source for producing these metabolites. However, the control of *H. contortus* with toxins derived from mushrooms or another natural alternative should not be considered as a unique option. A combination of several strategies must be carried out to avoid the development of any type of resistance or immunity by the parasite.

## Figures and Tables

**Table 1:** Challenge of *H. contortus* larvae L3 with *P. eryngii*.

Extract or control	*Pleurotus eryngii* strain (treatment)	Concentration	*H. contortus* larvae
AE	ECS-1138	20 *μ*g/mL	100 L3 in 80 *μ*L of distilled water
	ECS-1292		
	ECS-1258		
	ECS-1156		
	ECS-1255		
	ECS-1261		
	ECS-1282		
HA	ECS-1138	20 *μ*g/mL	
	ECS-1292		
	ECS-1258		
	ECS-1156		
	ECS-1255		
	ECS-1261		
	ECS-1282		
MeOH^1^	—	4%	
IVM^2^	—	10 mg/mL	

*n* = 5. ^1^Methanol (negative control); ^2^IVM (positive control).

**Table 2:** Death rate of *H. contortus* L3 larvae against HA and AE from *P. eryngii*.

Treatments	Hydroalcoholic extracts		Acetonic extracts	
	% of mortality ± sd	Group	% of mortality ± sd	Group
ECS-1255	18.83 ± 3.49	b	9.63 ± 2.44	b
ECS-1258	17.67 ± 5.73	bc	1.95 ± 2.47	cd
ECS-1156	15.46 ± 5.62	bcd	3.72 ± 2.82	cd
ECS-1292	13.64 ± 5.37	bcd	0.76 ± 2.02	d
ECS-1261	12.65 ± 7.66	cd	3.54 ± 3.53	cd
ECS-1138	12.47 ± 2.71	cd	1.50 ± 2.47	cd
ECS-1282	11.55 ± 5.69	d	2.30 ± 2.95	cd
IVM (10 mg/mL)	100 ± 00	a	95.56 ± 0.00	a
MeOH	0 ± 00	e	4.43 ± 0.91	c

*n* = 5, 72 hours postconfrontation 28°C. Groups with different letters indicate significant difference (*p* < 0.05) ANOVA and LSD test.

**Table 3:** Effectiveness of the F5 fraction on inhibiting the egg hatching of *Haemonchus contortus*.

Treatments	% egg hatching inhibition ± sd	Group
3 mg/mL	42.78 ± 3.84	c
5 mg/mL	48.56 ± 9.67	c
10 mg/mL	73.53 ± 12.34	b
20 mg/mL	88.76 ± 1.19	a
40 mg/mL	91.87 ± 3.35	a
IVM (5 mg/mL)	95.95 ± 0.00	a
DMSO 2.5%	1.01 ± 1.22	d

Treatments with different letters denote a significance; difference according to the Tukey test (*α* = 0.05).

## Data Availability

The data used to support the findings of this study are available from the corresponding author upon request.

## References

[1] Emery D. L., Hunt P. W., Le Jambre L. F. (2016). *Haemonchus contortus*: the then and now, and where to from here?. *International Journal for Parasitology*.

[2] Wang T., Van Wyk J. A., Morrison A., Morgan E. R. (2014). Moisture requirements for the migration of *Haemonchus contortus* third stage larvae out of faeces. *Veterinary Parasitology*.

[3] Laing R., Kikuchi T., Martinelli A. (2013). The genome and transcriptome of *Haemonchus contortus*, a key model parasite for drug and vaccine discovery. *Genome Biology*.

[4] Rodríguez-Vivas R. I., Grisi L., Pérez de León A. Á. (2017). Potential economic impact assessment for cattle parasites in Mexico. Review. *Revista Mexicana de Ciencias Pecuarias*.

[5] Medina P., Guevara F., La M., Ojeda N., Reyes E. (2014). Resistencia antihelmíntica en ovinos: una revisión de informes del sureste de México y alternativas disponibles para el control de nemátodos gastrointestinales. *Pastos y Forrajes*.

[6] Li G., Zhang K., Xu J., Dong J., Liu Y. (2007). Nematicidal substances from fungi. *Recent Patents on Biotechnology*.

[7] Ganeshpurkar A., Bhadoriya S. S., Pardhi P., Rai G. (2012). Investigation of anthelmintic potential of oyster mushroom *Pleurotus florida*. *Indian Journal of Pharmacology*.

[8] Hugo L. A. G., de Freitas Soares Filippe E., de Queiroz Joss H. (2015). Activity of the fungus *Pleurotus ostreatus* and of its proteases on *Panagrellus* sp. larvae. *African Journal of Biotechnology*.

[9] Li G.-H., Zhang K.-Q., Zhang K.-Q., Hyde K. D. (2014). Nematode-toxic fungi and their nematicidal metabolites. *Nematode-Trapping Fungi*.

[10] Stajić M., Vukojević J., Duletić-Lausević S. (2009). Biology of *Pleurotus eryngii* and role in biotechnological processes: a review. *Critical Reviews in Biotechnology*.

[11] Samsam Shariat H., Farid H., Kavianpour M. (1994). A study of the anthelmintic activity of aqueous extract of *Pleurotus eryngii* on *Symphacia* obvelata and *Hymenolepis nana*. *Journal of Sciences, Islamic Republic of Iran*.

[12] Lopes A. D. C. G., Hiura E., Soares F. E. F. (2015). Predatory activity of the fungus *Pleurotus eryngii* on *Ancylostoma caninum* infective larvae. *SOJ Veterinary Sciences*.

[13] Li G. H., Dong J., Mo M., Zhang K. (2001). Nematicidal activity of nematophagous *Pleurotus* and allied fungi to *Panagrellus redivivus*. *Chinese Journal of Biological Control*.

[14] Mamiya Y., Hiratsuka M., Murata M. (2005). Ability of wood-decay fungi to prey on the pinewood nematode, *Bursaphelenchus xylophilus* (Steiner and Buhrer) Nickle. *Nematological Research (Japanese Journal of Nematology)*.

[15] Palizi P., Goltapeh E., Pourjam E., Safaie N. (2009). Potential of oyster mushrooms for the biocontrol of sugar beet nematode (*Heterodera schachtii*). *Journal of Plant Protection Research*.

[16] Sánchez J. E., Royse D., Sánchez J. E., Royse D. J. (2001). El cultivo de *Pleurotus* spp. *La Biología y el Cultivo de Pleurotus spp. pp. 187–201.*.

[17] von Son-de Fernex E., Alonso-Díaz M. Á., Mendoza-de Gives P. (2015). Elucidation of *Leucaena leucocephala* anthelmintic-like phytochemicals and the ultrastructural damage generated to eggs of *Cooperia* spp. *Veterinary Parasitology*.

[18] Pineda-Alegría J. A., Sánchez-Vázquez J. E., González-Cortazar M. (2017). The edible mushroom *Pleurotus djamor* produces metabolites with lethal activity against the parasitic nematode *Haemonchus contortus*. *Journal of Medicinal Food*.

[19] Liébano-Hernández E., López-Arellano M. E., Mendoza-de Gives P., Aguilar-Marcelino L. (2011). *Manual de diagnóstico para nematodos gastrointestinales en rumiantes*.

[20] Belemlilga-Bonewendé M., Traoré A., Ouédraogo S., Kaboré A., Hamadou-Tamboura H., Pierre-guisoou I. (2016). Anthelmintic activity of *Saba senegalensis* (A.DC.) Pichon (Apocynaceae) extract against adult worms and eggs of *Haemonchus contortus*. *Asian Pacific Journal of Tropical Biomedicine*.

[21] Kitson F. G., Larsen B. S., McEwen C. N. (1996). Sugars (monosaccharides). *Gas chromatography and mass spectrometry. A practical guide*.

[22] Comans-Pérez R. (2014). *Evaluación de la actividad nematicida in vitro del micelio de diez hongos comestibles en contra del nematodo Haemonchus contortus L3*.

[23] Huicochea Medina M. (2015). *Actividad antihelmíntica in vitro del micelio de los hongos comestibles Pleurotus ostreatus (ECS-1123) y Pleurotus eryngii (ECS-1292) en contra de larvas infectantes (L3) del nemátodo parásito de ovinos Haemonchus contortus*.

[24] Li X., Feng T., Zhou F. (2015). Effects of drying methods on the tasty compounds of *Pleurotus eryngi*i. *Food Chemistry*.

[25] Eguale T., Gidey M., Mekonnen Y., Animal N., Ababa A. (2006). *In vitro* anthelmintic activities of four Ethiopian medicinal plants against *Haemonchus contortus*. *Pharmacology*.

[26] Eguale T., Tilahun G., Debella A., Feleke A., Makonnen E. (2007). *Haemonchus contortus* : *in vitro* and *in vivo* anthelmintic activity of aqueous and hydro-alcoholic extracts of *Hedera helix*. *Experimental Parasitology*.

[27] Harder A. (2016). *The Biochemistry of Haemonchus Contortus and Other Parasitic Nematodes*.

[28] Marie-Magdeleine C., Mahieu M., Archimède H., Preedy V. R., Watson R. R., y Patel V. B. (1999). Pumpkin (Cucurbita moschata Duchesne ex Poir.) Seeds as an Anthelmintic Agent?. *Nuts and Seeds in Health and Disease Prevention*.

[29] Perry R. N., Wharton D. A. (2011). *Molecular and Physiological Basis of Nematode Survival*.

[30] Lee L. D. (2002). Hatching. *The Biology of Nematodes*.

[31] Marie-Magdeleine C., Mahieu M., Archimède H. (2011). Chapter 110 - Pumpkin (*Cucurbita moschata* Duchesne ex Poir.) seeds as an anthelmintic agent?. *Nuts and Seeds in Health and Disease Prevention*.

[32] Seo S., Kim J., Koh S., Ahn Y., Park I. (2014). Nematicidal activity of natural ester compounds and their analogues against pine wood nematode, *Bursaphelenchus xylophilus*. *Journal of Agricultural and Food Chemistry*.

[33] Horie Y., Kanda T., Mochida Y. (2000). Sorbitol as an arrester of embryonic development in diapausing eggs of the silkworm, *Bombyx mori*. *Journal of Insect Physiology*.

[34] Ko M. P., Van Gundy S. D. (1988). An alternative gelling agent for culture and studies of nematodes, bacteria, fungi, and plant tissues. *Journal of Nematology*.

[35] Cedillo-Rodríguez C. (2016). *Estudio químico-biodirigido del extracto hidroalcohólico del hongo Pleurotus ostreatus con actividad nematicida contra Haemonchus contortus*.

[36] Kumar R., Singh S., Singh O. V. (2008). Bioconversion of lignocellulosic biomass: biochemical and molecular perspectives. *Journal of Industrial Microbiology and Biotechnology*.

[37] Nilsson K., Sangster M., Gallis C. (2011). *Forests, Trees and Human Health*.

[38] Park I.-K., Kim J., Lee S.-G., Shin S.-C. (2007). Nematicidal activity of plant essential oils and components from ajowan (*Trachyspermum ammi*), allspice (*Pimenta dioica*) and litsea (*Litsea cubeba*) essential oils against pine wood nematode (*Bursaphelenchus xylophilus*). *Journal of Nematology*.

[39] Deepak M., Dipankar G., Prashanth D., Asha M. K., Amit A., Venkataraman B. V. (2002). Tribulosin and *β*-sitosterol-D-glucoside, the anthelmintic principles of *Tribulus terrestris*. *Phytomedicine*.

[40] Ohri P., Pannu S. K. (2009). Effect of terpenoids on nematodes: a review. *Journal of Environmental Research And Development*.

[41] Hrckova G., Velebny S. (2013). Parasitic helminths of humans and animals. *Pharmacological potential of selected natural compounds in the control of parasitic diseases*.

[42] Degenkolb T., Vilcinskas A. (2016). Metabolites from nematophagous fungi and nematicidal natural products from fungi as alternatives for biological control. Part II: metabolites from nematophagous basidiomycetes and non-nematophagous fungi. *Applied Microbiology and Biotechnology*.

[43] Stadler M., Mayer A., Anke H., Sterner O. (1993). Fatty acids and other compounds with nematicidal activity from cultures of basidiomycetes. *Planta Medica*.

[44] Ghisalberti E. L., Atta-ur-Rahman (2002). Secondary metabolites with antinematodal activity. *Studies in Natural Products Chemistry*.

[45] Nagase A., Kuwahara Y., Tominaga Y., Sugawara R. (1982). Nematicidal activity of akylamine against the pine wood nematode, *Bursaphelenchus lignicolus*. *Agricultural and Biological Chemistry*.

[46] Sangsopha W., Lekphrom R., Kanokmedhakul S., Kanokmedhakul K. (2016). Cytotoxic and antimalarial constituents from aerial parts of *Sphaeranthus indicus*. *Phytochemistry Letters*.

[47] Begum S., Ayub A., Shaheen Siddiqui B., Fayyaz S., Kazi F. (2015). Nematicidal triterpenoids from *Lantana camara*. *Chemistry & Biodiversity*.

[48] Ferheen S., Akhtar M., Nisar Ahmed A. (2011). Nematicidal potential of the *Galinsoga parviflora*. *Pakistan Journal of Scientific and Industrial Research Series B: Biological Sciences*.

[49] Wood I. B., Amaral N. K., Bairden K. (1995). World Association for the Advancement of Veterinary Parasitology (W.A.A.V.P.) second edition of guidelines for evaluating the efficacy of anthelmintics in ruminants (bovine, ovine, caprine). *Veterinary Parasitology*.

